# Base of Tongue Squamous Cell Carcinoma With Metastasis to the Mandibular Symphysis: A Case Report

**DOI:** 10.7759/cureus.50417

**Published:** 2023-12-12

**Authors:** William Montagne, Wanda Thi, Logan Lee, Oluwafunmilola T Okuyemi, Robert C Wang

**Affiliations:** 1 Otolaryngology - Head and Neck Surgery, University of Nevada Las Vegas School of Medicine, Las Vegas, USA; 2 Otolaryngology - Head and Neck Surgery, University of Nevada Reno School of Medicine, Reno, USA

**Keywords:** head and neck cancer, metastatic oropharynx cancer, oropharynx cancer, squamous cell carcinoma, base of tongue squamous cell carcinoma

## Abstract

Squamous cell carcinoma (SCC) is the most common malignancy of the oropharynx (OP). Treatment of OP SCC includes chemotherapy, radiation, and/or surgery. OP SCC can spread via direct extension, lymphatics, or hematogenously. Although rare, distant metastases can occur in OP SCC. The most common sites of metastasis include the lungs, bone, and liver. Other less common sites include the skin, bone marrow, brain, kidneys, eyes, and heart. Patients who present with distant metastases usually have a poor prognosis. Sites of bone metastases from more common to less common include the spine, skull, ribs, and axial bones. In this article, we discuss a patient who presents with HPV+ base of tongue SCC with metastases to the lungs and mandible symphysis. Base of tongue SCC metastasizing to the mandible symphysis is a rarely reported location of metastasis.

## Introduction

Squamous cell carcinoma (SCC) is the most common malignancy of the oropharynx (OP). SCC represents about 90% of OP malignancies and of those, about 25-40% involve the tongue [1-3]. Treatment of OP SCC includes chemotherapy, radiation, surgery, or a combination of these. Five-year survival of OP SCC is about 50% [1]. Oropharyngeal SCC can metastasize via direct extension, lymphatics, or hematogenously. Oropharyngeal SCC has aggressive local destruction and regional lymphatic spread. Distant metastasis is rare (4-26%). The most common sites of distant metastasis include the lungs, bone, and liver [1,2,4-7]. Other less common sites of metastasis for OP SCC include skin, bone marrow, brain, kidneys, eyes, and heart [1,3,8-10]. Patients with these distant metastases usually have a poor prognosis and are treated palliatively [9-11]. Although rare, metastases to bones occur. A list of bony metastatic sites from most common to least common includes the spine, skull, ribs, and axial bones [6]. To our knowledge, base of tongue (BOT) SCC metastasizing to the mandible is rarely reported beyond direct extension. Here we present a rare case of p16+/HPV+ SCC metastasizing to the mandibular symphysis. 

## Case presentation

**Figure 1:**
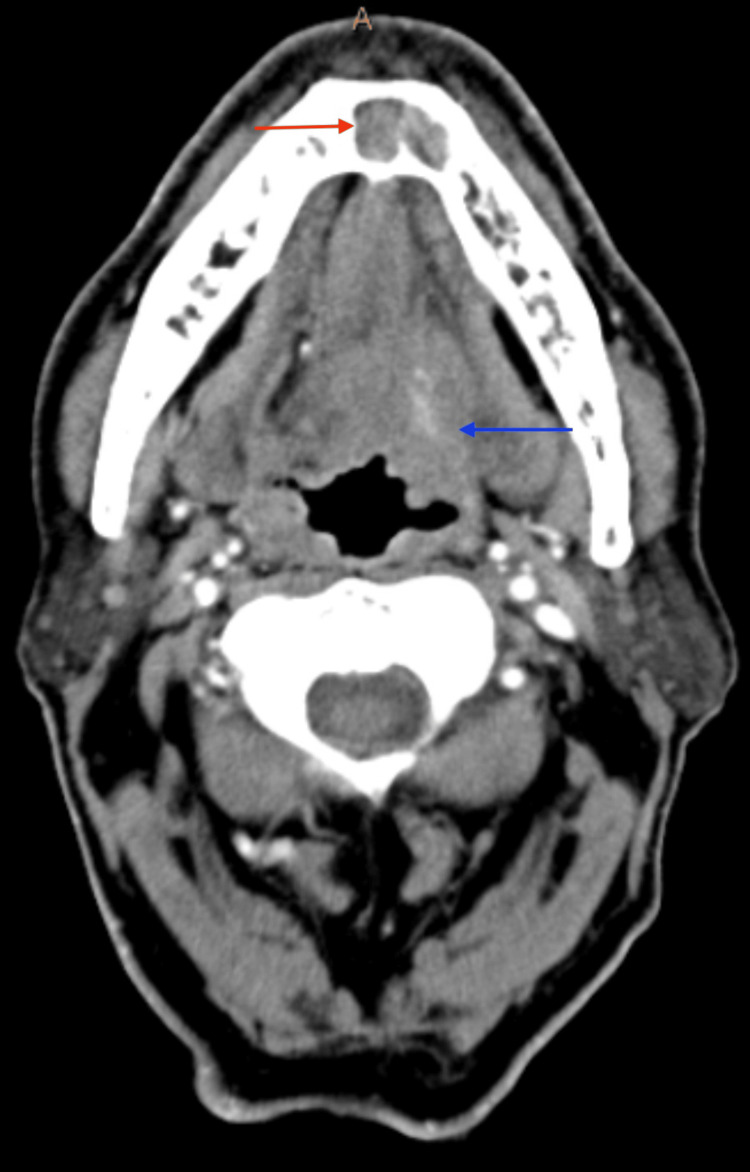
Axial CT Neck of Mandibular and Base of Tongue Mass Axial CT neck with contrast showing a soft tissue mass in the mandibular symphysis (red arrow) and a left base of tongue mass (blue arrow)

**Figure 2:**
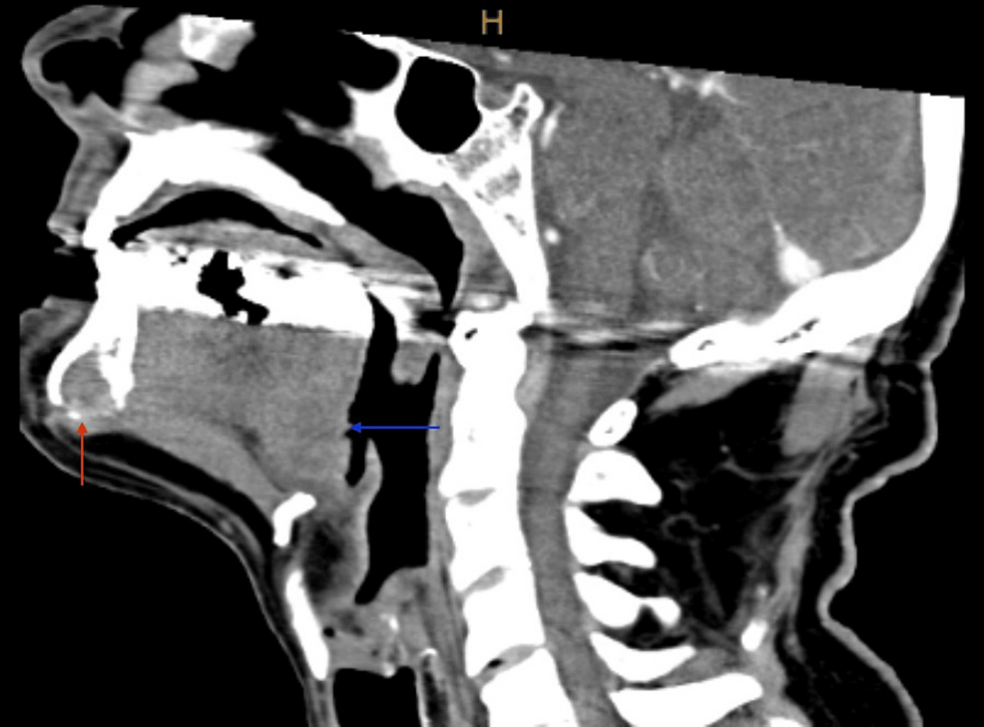
Sagittal CT Neck of Mandibular and Base of Tongue Mass Sagittal CT neck with contrast showing a soft tissue mass in the mandibular symphysis (red arrow) and a left base of tongue mass (blue arrow)

**Figure 3:**
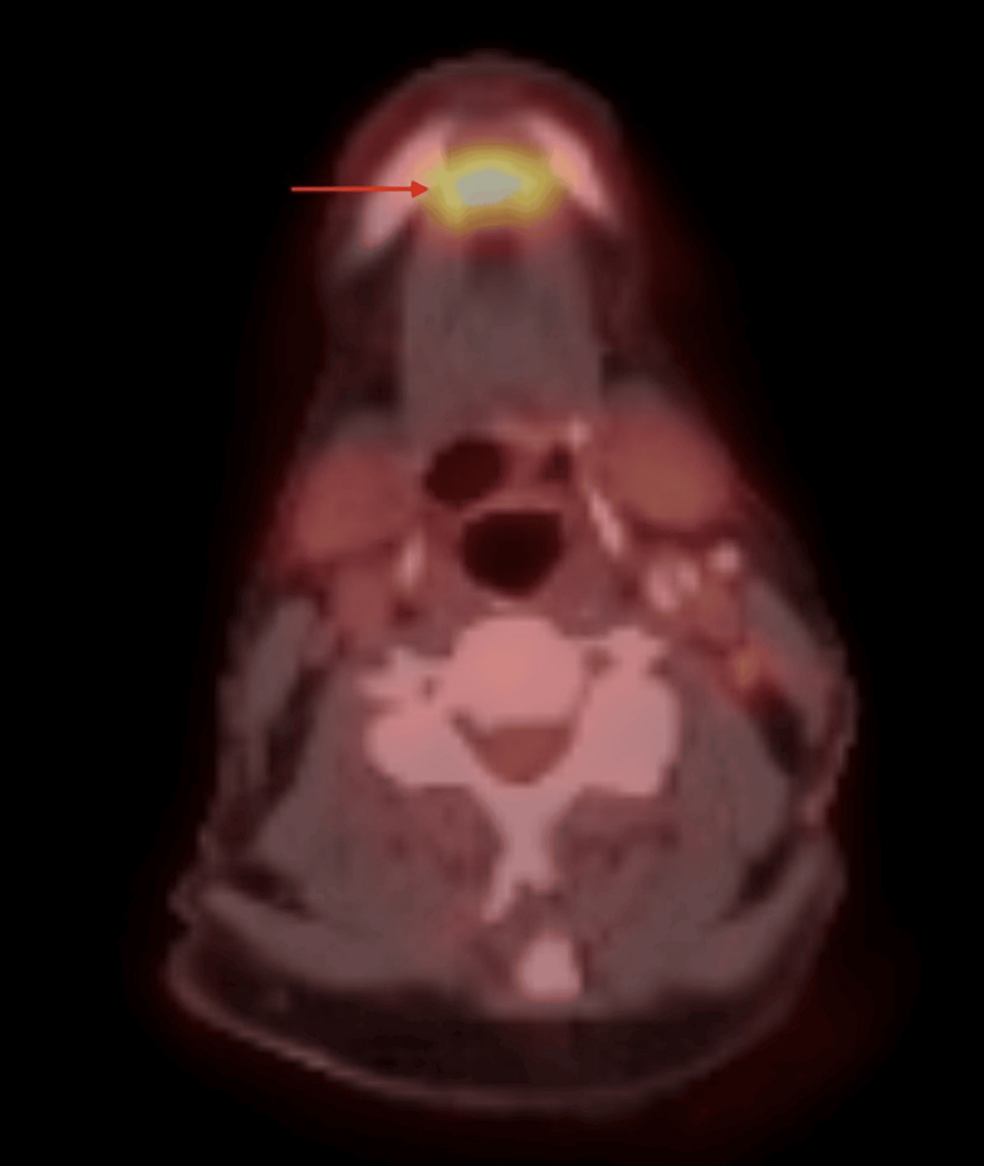
Axial PET/CT Neck of Mandibular Mass Axial PET/CT neck showing a FDG avid mass in the mandibular symphysis (red arrow)

**Figure 4:**
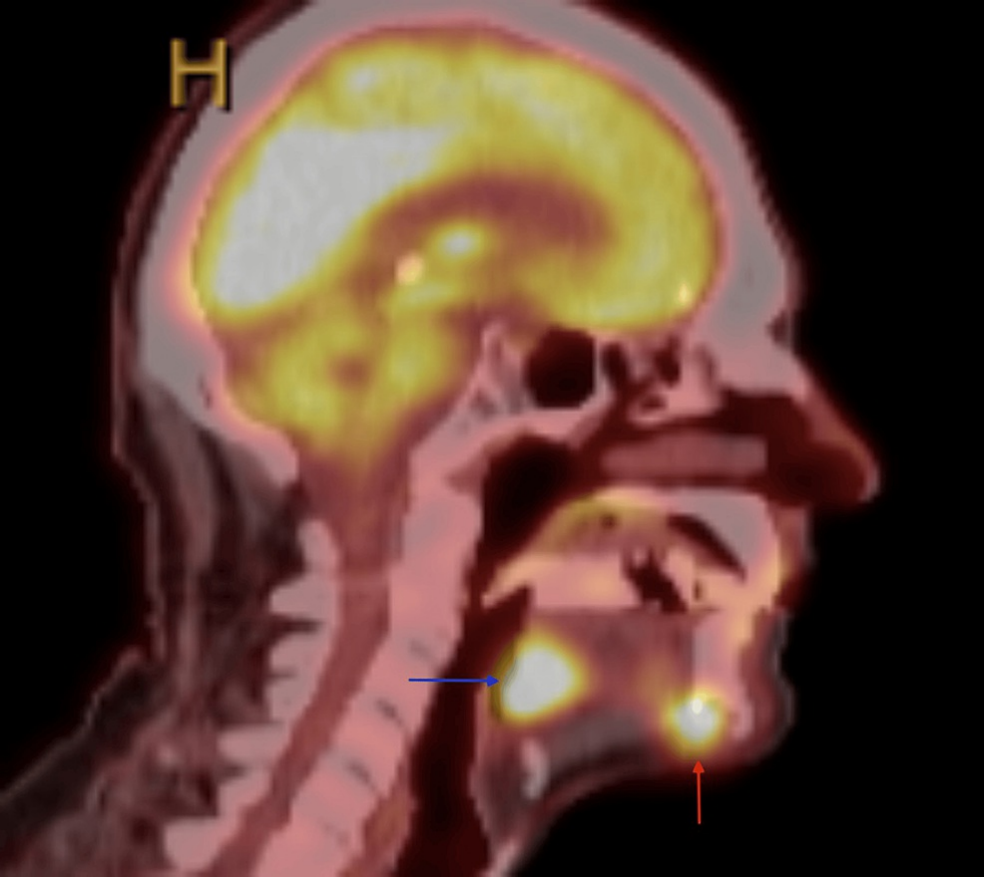
Sagittal PET/CT of Mandibular and Base of Tongue Mass Sagittal PET/CT neck showing FDG avid soft tissue mass in the mandibular symphysis (red arrow) and a left base of tongue mass (blue arrow)

**Figure 5:**
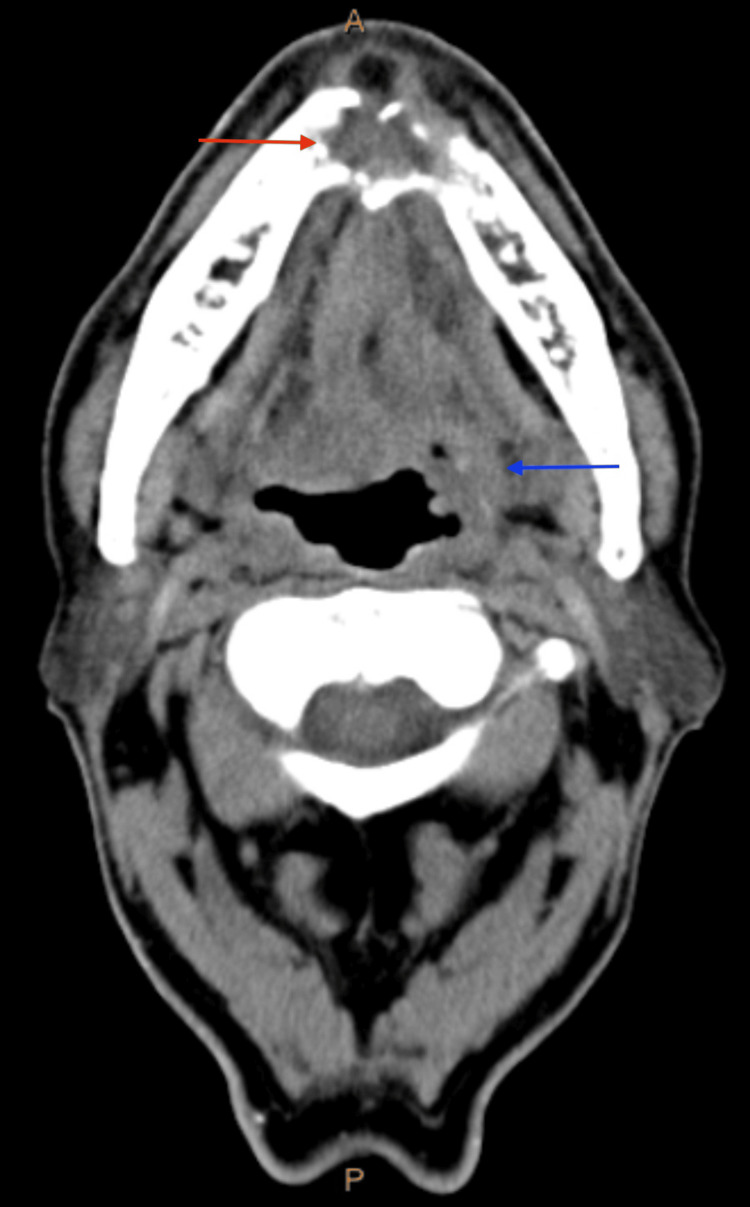
Post-induction Chemotherapy Axial CT Neck of Mandibular and Base of Tongue Mass Post induction axial CT neck with contrast showing worsening of soft tissue mass in the mandibular symphysis (red arrow) and decreased size of the left base of tongue mass (blue arrow)

**Figure 6:**
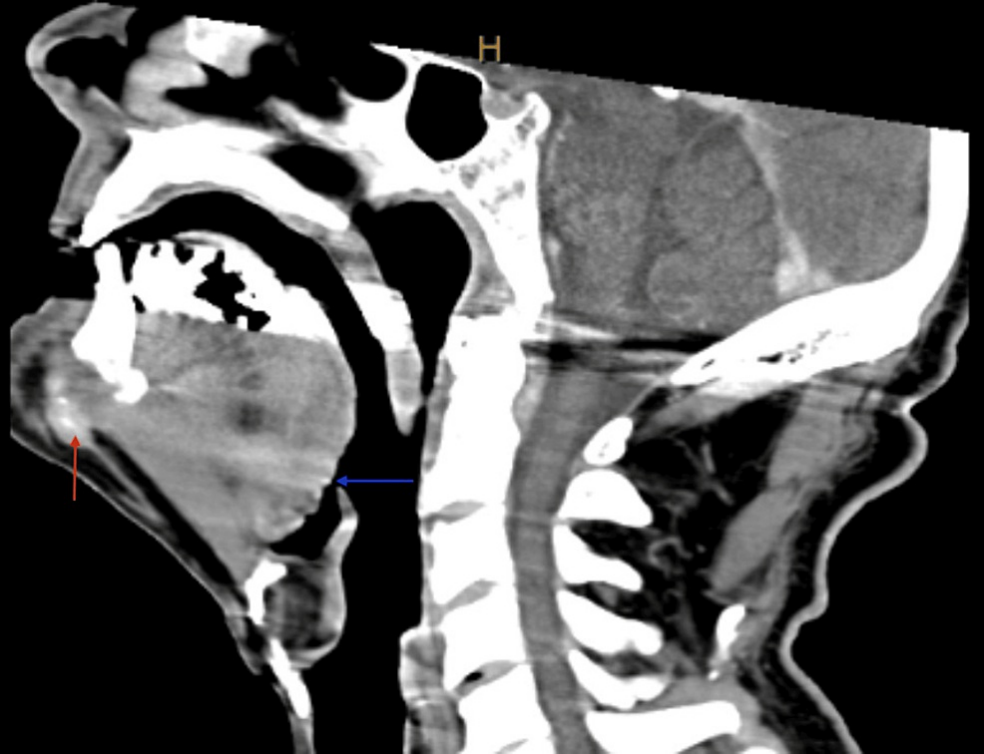
Post-induction Chemotherapy Sagittal CT Neck of Mandibular and Base of Tongue Mass Post induction sagittal CT neck with contrast showing worsening of soft tissue mass in the mandibular symphysis (red arrow) and decreased size of the left base of tongue mass (blue arrow)

A 75-year-old male with extensive smoking and drinking history presented to our clinic with a left BOT mass and left neck lymphadenopathy. His initial presentation was for a persistent left neck mass. Before his presentation to our clinic, a biopsy of his left neck mass was positive for SCC. He had imaging (including CT neck, CT chest, and PET scan) that showed the left base of tongue mass, bilateral lymphadenopathy, and probable lung metastasis. CT neck with contrast showed both the BOT and mandibular symphysis masses (Figures 1, 2). This was also seen on PET imaging with an FDG avid mass in the mandible symphysis and left BOT (Figures 3, 4). He was taken to the operating room about two weeks after his first visit for direct laryngoscopy and biopsy of the BOT. Pathology showed p16+ invasive SCC that was HPV 16/18 positive. He was started on induction chemotherapy one month after his first visit with docetaxel, cisplatin, and 5-FU. He completed three cycles of induction chemotherapy and had a repeat PET scan. The scan showed response at the BOT and lungs with persistence of the anterior mandible mass (Figures 5, 6). He was offered chemoradiation (ChemoXRT) vs surgery with adjuvant ChemoXRT. He selected the latter. Five months after his first visit, he underwent a composite resection of the left base of tongue with mandibulectomy, bilateral suprahyoid neck dissection, and reconstruction with a right osteocutaneous fibula free flap.

Original pathology for the left base of tongue biopsy was p16+ SCCa. The composite resection of the mandible showed p16+ SCCa with lymphovascular invasion and perineural invasion. Right level 1A and B and left level 1B neck dissection showed no nodal involvement. 

Post-operative PET and CT chest showed FDG uptake at the left BOT and scattered pulmonary nodules. He started chemoXRT and completed 35 sessions of radiation. Imaging after completing chemoXRT showed persistent/enlarging pulmonary nodules and residual vs new midline BOT mass. He started immunotherapy (cetuximab), without improvement. Further imaging showed worsening of disease with metastasis to the ribs. He underwent radiation therapy to the chest cavity for these. His condition continued to deteriorate, and he succumbed to his disease about 22 months after diagnosis of SCC.

## Discussion

SCC is a common form of OP malignancy. SCC of the oropharynx can present with aggressive direct extension or lymphatic metastases. Distant metastases in oropharyngeal SCC are rare. The most common sites of distant metastasis include the lungs, bone, and liver [1,2,4-7]. Less common sites of metastasis for OP SCC include skin, bone marrow, brain, kidneys, eyes, and heart [1,3,8-10]. Above we presented a patient with advanced p16+/HPV-positive OP SCC who had metastases to the lungs and mandibular symphysis. To our knowledge, oropharyngeal SCC with metastasis to the mandible symphysis is rarely reported.

Oropharyngeal SCC was historically associated with alcohol and tobacco use. Now there is a rising incidence of high-risk HPV-positive OP SCC [12]. HPV-positive OP SCC is usually seen in younger patients with low exposure to tobacco and alcohol. Patients who present with HPV+ OP SCC usually have a lower T stage and higher nodal status. Previous research has shown that HPV status surpasses traditional prognostic markers (TNM staging) in prognostic strength [12]. With the rise in HPV+ OP SCC, there was a concern about patients being overtreated in HPV+ cases. AJCC 8th edition introduced a new staging paradigm for HPV+ OP SCC [13]. In OP SCC, p16 staining has been used as a surrogate marker of HPV status, but recent research has shown that HPV DNA and p16 provide superior prognostic information compared to p16 alone in tonsil and BOT OP SCC [14]. HPV status has not been shown to be as good as a prognostic marker for OP SCC outside the tonsil and BOT. HPV-positive OP SCC exhibits different patterns of recurrence compared to HPV- OP SCC. Both have rare late recurrence with HPV-negative more likely to recur locally and HPV+ having significantly higher distant recurrence [14]. 

Distant metastases in OP SCC are rare but do occur. About 30% of patients with lymph node involvement will have distant metastases that occur about 9-12 months after initial diagnosis [1]. The most common sites for OP SCC to metastasize include the lung, bones, and liver [1,2,4-7]. There have also been reports of other less common sites including the skin, kidneys, eyes, and heart [1,3,8,9,10]. Once distant metastases are discovered long-term survival is generally poor. Our patient above presented with metastases to the lungs and mandible. Mandibular involvement in head and neck SCC is usually through contiguous spread as lymphatics are sparse in this region and not considered a route of spread [15]. Although rare, bony metastasis does occur in head and neck SCC with sites from more to less common including the spine, skull, ribs, and axial bones [6]. As our patient presented with a BOT primary, we should consider his mandibular involvement as a distant metastasis. His CT findings likely correspond with this as the mass at the mandibular symphysis appears to grow from within the mandible and spread out vs direct extension.

This patient also has demographic and pathological data for both extensive smoking and alcohol use, as well as HPV+ pathology. This limits the ability to determine if HPV or more traditional causes of OP SCC may influence this rare site of metastasis. The patient also presented at a late stage with distant metastases and did not have imaging before the mandible was involved. Imaging that showed the start of the involvement and progression of the mandible could further confirm this as a metastasis over possible direct spread or second primary. Although we found sources that mention the skull as a possible site of metastasis, there was no mention of locations. A further study evaluating the subsites of metastasis to the skull could further determine how often the mandible may be involved.

## Conclusions

Overall, we presented a patient with advanced BOT SCC with distant metastases to the lungs and mandible. OP/BOT SCC metastasizing to the mandible is rarely reported. This is important to consider as involvement of the mandible can influence treatment and quality of life in head and neck cancer patients. Once patients with OP SCC develop distant metastasis, outcomes are generally poor. Further research into sub-locations of skull metastasis of OP SCC may help with understanding prognosis and best treatment pathways in these patients.
